# A sentence sliding window approach to extract protein annotations from biomedical articles

**DOI:** 10.1186/1471-2105-6-S1-S19

**Published:** 2005-05-24

**Authors:** Martin Krallinger, Maria Padron, Alfonso Valencia

**Affiliations:** 1 Protein Design Group, National Center of Biotechnology, CNB-CSIC, Cantoblanco, E-28049 Madrid, Spain

## Abstract

**Background:**

Within the emerging field of text mining and statistical natural language processing (NLP) applied to biomedical articles, a broad variety of techniques have been developed during the past years. Nevertheless, there is still a great ned of comparative assessment of the performance of the proposed methods and the development of common evaluation criteria. This issue was addressed by the Critical Assessment of Text Mining Methods in Molecular Biology (BioCreative) contest. The aim of this contest was to assess the performance of text mining systems applied to biomedical texts including tools which recognize named entities such as genes and proteins, and tools which automatically extract protein annotations.

**Results:**

The "sentence sliding window" approach proposed here was found to efficiently extract text fragments from full text articles containing annotations on proteins, providing the highest number of correctly predicted annotations. Moreover, the number of correct extractions of individual entities (i.e. proteins and GO terms) involved in the relationships used for the annotations was significantly higher than the correct extractions of the complete annotations (protein-function relations).

**Conclusion:**

We explored the use of averaging sentence sliding windows for information extraction, especially in a context where conventional training data is unavailable. The combination of our approach with more refined statistical estimators and machine learning techniques might be a way to improve annotation extraction for future biomedical text mining applications.

## Background

Experimental results related to molecular biology research are published in the form of journal articles and stored in biomedical literature databases such as PubMed [1]. The amount of scientific articles is increasing dramatically. Nonetheless, useful information in terms of functional descriptions of proteins and genes, is still extracted from these publications manually by human experts. The extracted information is consequently used to build up annotations within biological databases describing relevant aspects of these proteins. These annotations are also commonly used to automatically infer annotations to other, sequence-related proteins, as often protein sequence similarity can give clues to functional similarity. This automatic sequence-based annotation in some cases can be misleading as even the conservation of function itself is often difficult to estimate [2,3]. As the amount of feasible annotations using manual information extraction is limited, it may not keep up with the pace of article publication and thus represents a severe bottleneck preventing knowledge gain. Current estimates are that 10% of protein sequences are annotated from original sources with the remaining 90% being inferred from that 10%.

Efficient automatic filtering and information extraction algorithms for biomedical literature are needed to compliment manual information extraction and reduce the human effort needed. Moreover, they could aid in maintaining links from database annotations to article sources. In order to speed up the annotation process, a broad variety of methods drawn from the field of text mining and statistical natural language processing (NLP) have been applied to common biological problems. Some techniques have been applied directly to derive important information for protein annotations [4-6]. Other approaches have been intended to complement the analysis of microarrays [7-10] in terms of the biological background information of the analyzed genes. Attempts have also been made to automatically extract protein interactions [11] and to improve protein sequence similarity searches [12,13]. Although a considerable number of text mining methods applied to biomedical articles are currently available, common assessment initiatives were missing. This has made it especially cumbersome to compare the relative strengths of different techniques so as to improve future applications.

We present a first version of our approach to extract function annotation passages from free full text articles. This procedure produced the highest number of correct annotation extractions in the BioCreative contest (task 2.1) [14]. The BioCreative is a community wide experiment with the purpose of assessing distinct techniques of named entity recognition and gene tagging (tasks 1a and 1b) [15,16] as well as automatic protein annotation extraction (tasks 2a and 2b) [14,17].

BioCreative task 2.1, was concerned with the automatic extraction of protein annotations. The evaluation was carried out by expert annotation database curators from the EBI GOA team [17,18], providing a high quality assessment. In this task, the entities involved in the annotation, a protein identifier and a Gene Ontology (GO) code were provided. GO is a dataset containing consistent descriptions of gene products in the form of controlled vocabulary terms. It consists of an ontology with a directed acyclic graph structure. Each entry may belong to one of three categories, molecular function, biological process or cellular component and has a unique associated GO code. For each GO code and protein-identifier pair (entities), given a full text article, the text passage which would be useful to derive a GO-based protein annotation (relationship) should be returned. Those text fragments should thus contain traceable associations between the protein entity and the GO-term entity.

In order to extract the required annotation passages, lists of terms and word types for entities involved in the annotation were compiled. For each list, a semi-heuristic scoring scheme was developed, which was used to score sentences after tagging them with the elements recovered from each list. Those scores were then used by averaging sentence sliding windows to score each sentence taking into account the context of flanking sentences. The highest scoring sentence window was returned as the annotation evidence text passage.

## Materials and Methods

### Gene Ontology Annotation dataset

In order to analyze the associations of proteins with GO-terms within scientific articles we used the Gene Ontology Annotation database (GOA) [18]. This database provides annotations of proteins using GO-terms through associations derived from the scientific literature. For instance for the SwissProt annotation database accession number 'O00115' (SWISS – PROT/TrEMBL entry name DRN2_HUMAN) the GO identifier 'GO : 0003677' was annotated using information contained in PubMed document with the PubMed identifier 9714827. We extracted a dataset of 560 human protein- GO-term annotations contained in GOA and compiled the PubMed abstracts relevant for annotation as our "training" dataset. Only GOA database annotations with traceable author statements, as provided by the GOA-TAS evidence code were used. Traceable author statement annotations refer to annotations in which the original experiments are traceable through the article by a corresponding author statement. Those abstracts served for further statistical analysis of the sub-tag sets of each annotation entity. This dataset was rather noisy.

### Protein entity tag set

In order to identify functional annotations for proteins, information extraction of text passages relevant to them is crucial. As proteins constituted one of the entities involved in the annotation, we constructed a tool which generates lists of sub-tag sets for a given query protein. Each sub-tag set consisted of a list of word types and names associated with the protein query (see table 3), characterized by a distinct degree of relation with the original query protein.

Among the sub-tag sets used for the protein entity class were the original protein name, symbol or identifier, e.g. the SwissProt accession number. In the case of this contest the SwissProt accession number was provided as the protein query, consisting of a unique identifier for each protein entry in the SwissProt database (e.g. *O00115*) [19]. Within textual sources, protein symbols or names are often expressed in the form of different typographical variants [20]. Therefore we derived another sub-tag set containing protein variants which were generated through a rule based pipeline of protein name processing (in the example *O00115*: *DNASE2*, *DNASE 2 *and *DNASE-2*). Often different names for a certain protein, (synonyms) are obtained through cross references to other biological databases.

We developed a database which contained cross linked protein entries derived from several sources, including the HUGO, OMIM, SwissProt, UniGene and LocusLink databases. This database allowed us to extract all the possible naming conventions and definitions for a query protein based on its database identifier. All the protein symbols and names obtained through external links were also incorporated into a separate sub-tag set. For the example presented above, the elements contained in this sub-tag set included: '*deoxyribonuclease II*, *lysosomal*', '*DNL*' and '*DNL2*' (from HUGO). The word types forming the gene names also constituted a sub-tag set. For instance, in the case of O00115 the following word types were part of this sub-tag set: '*deoxyribonuclease*', '*II*', '*lysosomal*'. The pragmatic context information was also exploited as a sub-tag set to take into account the meronymic relations (whole-part relations) for proteins. Hence we used terms contained in the Global Open Biology Ontologies (GOBO) dataset. GOBO contains structured sets of terms related to different aspects within the field of molecular biology. We used the mutation event and sequence ontology tables, and exploited them as a separate sub-tag set. The presence of such meronymic terms might aid in disambiguating certain protein symbols using context information. Examples of the "GOBO mutation event sub-tag set" were *mutation *(0000128) and *conformational change *(0000116) and of the "GOBO sequence ontology sub-tag set" were *EST *(SO:0000345) and *transcript *(SO:0000673).

### Gene Ontology term tag set

As already mentioned GO-terms are controlled vocabulary items embedded into an ontological structure. To determine if these terms are suitable for NLP tasks, the lexical properties of GO [21] were analyzed. This analysis revealed that most of the GO-terms were useful for NLP approaches and some of them are even rather often encountered in free text. Nevertheless after a closer look at the GO-terms, we decided to construct a sub-tag scheme by analogy to the gene/protein entity (see table 3). Some of the GO-terms, especially those which denote more specific features, do not resemble what one would expect in free text. Hence it would be rather cumbersome to tag them in biomedical texts. Also certain terms within the categories molecular function and cellular component did not seem to correspond to natural language expressions due to the presence of special characters such as the backslashes. Some terms indicated the organism source which in principle should not form part of the term itself (e.g. *sensu Animalia*). From a linguistic point of view, a significant difference between protein symbols/names and the GO-terms is that the former are proper nouns while the latter can be considered to be adverbial nouns. Therefore, the GO-terms are even more diffcult to identify in free text as they often lack morphological characteristics which are present in proper nouns, such as capital letters or special characters as in the case of gene names (e.g. Greek letters). To recover some of the GO-terms it seemed therefore crucial to process them so that they would resemble their *natural language (NL) variants*, namely how they might be encountered in free text. We developed for this purpose a rule based system which modifies an input GO-term returning several potential NL-variants. Some of the processing steps performed by this system were minor typographical changes (e.g. lower case conversion) and word token substitutions by corresponding synonyms or adjective into noun conversions (e.g. *via *- >*through *and *viral *- >*virus*. Other processing events include acronym expansion (e.g. *ER *- >*endoplasmatic reticulum*), collocation shuffing and preposition insertions (e.g. *of *- >*of the*. Sample NL-variants for the GO-term *ER membrane viral budding *(GO:0046764) are *ER membrane virus budding *and *endoplasmatic reticulum membrane viral budding *and for *condensed nuclear chromosome/perocentric region *(GO:0000780) one of the NL-variants is *pericentric region of condensed nuclear chromosome*. Among the resources provided by the Gene Ontology consortium were also a GO-term synonym list and links for GO-terms to external databases such as the MIPS database keywords [22]. These synonyms and externally linked keywords were included as a separate tag set. Finally the word types from which GO-terms and the GO-term definitions (after extensive stop word filtering) are formed, were included in two respective tag sets, e.g. for the GO-term *regulation of mitotic recombination *(GO:0000019), the word types '*regulation*', '*mitotic*' and '*recombination*' were present in the sub-tag set containing GO-term word types. We generated stemmed versions for all the tag sets using the Porter Stemmer [23].

### Analysis of sub-tag sets using GOA abstracts

After defining the different sub-tag sets for each entity, we determined their utility for extracting annotation passages. Therefore we analyzed first the average number of occurrences for each sub-tag using GOA abstracts (see figure 3). As already pointed out this dataset was not representative enough in context of annotation text fragments and thus did not constitute a conventional training set. Moreover the total number of studied GOA abstracts was small and noisy, so it did not satisfy all the needed criteria for a representative annotation text sample. Nevertheless, we considered that the average occurrence of each sub-tag reflects somehow its specificity as an evidence item. We thus derived a heuristic weighting factor (heuristic sub-tag score *h*_*i*_) associated with each sub-tag which depended on the type of relationship it displayed with the original query and its own average occurrence. More specific sub-tags (e.g. the original GO-term or the gene symbol) were given a higher weighting score than more general sub-tags (e.g. the word types forming the GO-term or protein name). The stemmed versions of each sub-tag were scored lower relative to the original (unstemmed) word/s (as stemmed versions may result in ambiguous words). In the case of the word types forming the GO-term, GO-term definitions and composed protein names, an extended stop word filtering was performed. We started using several different heuristic weights for each sub-tag. Then, using a small set of sample articles, we adjusted the weights until the correct text passages (which contained the annotation) were returned as the highest scoring text window.

Let *h*_*i *_be the heuristic sub-tag score (weight) for each sub-tag *i *for a given entity, then *h*_*i *_would be calculated by:



where  is the average number of occurrences of elements of sub-tag *i *in GOA sentences and *e*_*i *_corresponds to the relative heuristic weight used for sub-tag *i*, based on domain expert estimates, the relation to the query term and finally some adjustments based on a small sample of full text articles containing an annotation passage. Notice that the heuristic sub-tag weights for the GO-term entity were in general higher than for the protein entity.

### Low level document processing and instantiation of sub-tag elements

The test set provided for task 2a consisted in full text articles from the Journal of Biological Chemistry in *SGML *format. The first step consisted in previous SGML-parsing, low-level processing and junk formatting. All the analyzed documents were subjected to a rule-based sentence splitting algorithm. Section and paragraph information was retained in the form of empty sentences (this is important for the sliding window procedure explained later). After performing the low-level processing, we tagged to the document sentences the lists of word types and terms contained in the sub-tag sets for each entity, using an exact string matching algorithm. Thus only the sub-tag elements which are matched to a sentence are instantiated. Each sentence has thus a set of GO entity and Protein entity sub-tag elements which were encountered within this sentence. There are also sentences with no matches to sub-tag elements, for instance the empty sentences corresponding to sections and paragraphs. In the case of the following sentence the sub-tag it Golgi corresponding to the sub-tag class containing GO-term forming words was machted: *Once fully glycosylated, the enzyme is phosphorylated and released from membranes either in or after the trans-Golgi compartment (*<*BBR RID="B12"*>).

### Trapezoid sentence sliding window

The use of the concept *sliding window *spans a broad variety of domains, such as information technology where it has been widely used in signal processing for analysis of frequent items in packet streams [24]. In bioinformatics, it has been applied to protein sequence analysis such as the prediction of transmembrane protein segments and to generate protein hydropathy profiles [25]. Sliding windows have also been applied within the field of natural language processing for collocation detection [26]. In our case we explored the use of averaging sliding windows for information extraction tasks. We applied a trapezoid sentence sliding window to extract relevant text fragments for biomedical entities (proteins and GO-terms) using biomedical literature. The sliding window unit consists of sentences.

Let L be the total number of sentences forming the trapezoid sliding window (window length), then the sentence position weight *w*_*i *_within the window was determined by



In this way, we scored the flanking sentences comprising the sliding window lower then the core sentences of the window. This is based on the assumption that the flanking sentences might contain contextual information useful for scoring the sentences relative to the presence of a given entity.

The average sliding sentence window entity score,  was calculated by



where *w*_*i *_is the corresponding sentence position weighting factor and L is the sentence window size. In the case of the entity profiles, L = 5 sentences. *S*_*i *_is the sum of the scores of the matching sub-tags of a given sentence, n being the total number of matched items and *h*_*i *_the associated heuristic sub-tag score.



### Entity profiles

The "trapezoid sliding windows" generate average entity scores for each sentence within the document. Taking the average sentence scores relative to the sentence number, it is possible to generate a document entity profile for the GO-term as well as for the protein. The higher the average sentence score, the more likely it should be that the corresponding text window contains information relative to the entity or to items associated with the entity. These profile scores were used to determine relevant text passages for each entity. The values of the scores can hint at the types of sub-tags being matched to the window, as high average window scores are associated with high scoring sub-tag matches. In general the average sentence scores for proteins are lower then for GO-terms, this is due to the overall weighting scheme used for the sub-tags.

### Annotation profile

After calculating the average entity score for each sentence we had to combine both resulting entity document profiles into a unique annotation document profile (see figure 4). The annotation profile should score every sentence window on whether it contains suitable information for annotating proteins with GO-terms. This was achieved using a combining sliding window which, as for the entity sliding windows, averages the sentence scores over a certain window size. The sliding window size used to generate the annotation profile was reduced to L = 4 sentences, as larger windows would result in text fragments too cumbersome to be evaluated by the assessor. The sentence position weights *w*_*i *_used in the case of the entity windows were ignored, meaning the flanking sentences had the same weighting as central sentences.

We therefore assumed that semantic information expressing the relation between two entities should be restricted to a distance expressed in sentences.

To calculate the average annotation sentence score , the entity profile sentence scores were used. The average annotation score  for a given sentence window is given by:



where L = 4 (window size), *(GO) *is the average entity sentence score for the GO-term and *(Protein) *the average entity sentence score for the protein. The sentence window with the the highest average annotation score A' was returned: A' = arg max  score(  ) as the annotation passage.

## Results

The proposed procedure derives individual sentence scores for each entity or class. It uses the weight scores of matched word tokens and entity terms. Each weight score depends on the type of tag (sub-tag) the matched token belonged to. Every sub-tag set consist of a list of word types or terms with a certain degree of association with respect to the initial query entity. The weight score itself for a given sub-tag is based on its occurrence in Gene Ontology Annotation (GOA) [18] abstracts and additional heuristic estimates. The sentence window then slides over the whole document generating two entity profiles by averaging over the summed sentence scores comprised in the window. This results in a *protein entity profile *and a *GO-term entity profile *when considering the *average entity sentence score *for each of the window positions. These profiles serve as input for a second averaging procedure which combines two entity-profiles to obtain a single document annotation profile using a sentence sliding window. The highest scoring sentence window was returned as the annotation evidence text.

We submitted a total of 1076 text passages as candidate predictions to provide relevant textual information for protein-GO-term annotations. Each segment used for annotation prediction consisted of four consecutive sentences extracted from the corresponding full text article. The curators evaluated 1050 of our submissions on whether they were relevant as traceable annotations (annotations which are based on concrete text segments). The assessment included separate evaluations of the extraction of each individual entity (proteins and GO-terms). Three distinct evaluation categories were proposed by the assessors. The category *perfect*, refers to correct predictions of the annotation textual passages, the category *general *refers to predictions that are 'in principle correct' but too general for practical use (i.e. a term belonging to a high level within the ontology) and finally the category *low *which in effect refers to a wrong prediction.

Due to the fact that a vast amount of data had to be evaluated, a minor fraction of submissions remained without assessment and were returned with the label *None*.

### Annotation extraction

We obtained the highest number of correct predictions of annotations for task 2a, with a total of 303 correct textual evidences (see table 1 and figure 5). This means 28.8% of submissions were correct. Nevertheless, with respect to precision, there were groups with a higher precision (figure 5), but they did not produce results for all the queries. There were 69 cases were the GO-term was correct and the protein extraction was general (6.57%). Moreover predictions with correctly predicted protein entities and general GO-term prediction constituted a total of 112 cases (10.67%). A sample annotation extraction which was evaluated as correct for SwissProt accession number 0005545 (endofin) and GO-id 0005545 (phosphatidylinositol binding) was:

In addition, a single point mutation in the FYVE finger motif at cysteine residue 753 (C753S) is sufficient to abolish its endosomal association. Its endosomal localization is also sensitive to the phosphatidylinositol 3-kinase inhib itor, wortmannin. Using in vitro liposome binding assays, we demonstrate that Myc-tagged endofin associates preferentially with phosphatidylinositol 3-phosphate, whereas the C753 S point mutant was unable to do so. We also show that endofin co-localizes with SARA but that they are not associated in a common complex because they failed to co-immunoprecipit ate in co-expressing cells.

As can be observed from above the example, the protein name and relevant word types for the GO-term are both contained within this textual passage.

### Entity extraction

With respect to the evaluation of the individual entity extractions, we obtained a total of 594 correct evidences for the protein entity, which corresponds to about 56% of total submissions (see table 1). This seems a satisfactory result, considering that we did not apply any anaphora (the use of pronouns instead of name repetitions) resolution algorithms and were only provided with the protein SwissProt identifier. We extracted a total of 501 (48%) correct GO-term evidences. Our system thus performed worse for GO-term extraction than protein entity extraction. For instance, the example presented above for annotation extraction was also extracted correctly for the protein entity *endofin *and the GO-term entity *phosphatidylinositol binding*.

### Annotation extraction relative to GO categories

The difficulty of predicting annotations varied with GO category (see table 2). Also, the extraction of GO-term entities themselves depended heavily on the associated GO category. The highest percentage of correct predictions of GO-terms came from the category "Cellular Component", followed closely by the "Molecular Function" category. Terms belonging to the "Biological Process" category were the most cumbersome to extract. This concurs with previous attempts to map terms to biomedical articles [27], that have also shown that recall was significantly lower for terms from the Biological Process category. This is explained by the fact that these terms are often expressed colloquially in different ways.

The ranking of correct GO-term extraction and of correct GO annotation extraction displays a shift in the case of the Cellular Component and the Molecular Function groups. The highest number of correct annotations corresponded to the category Molecular Function instead of Cellular Component. As word types forming Cellular Component terms are often used in other contexts (e.g. the word type "*membrane*" as part of experimental method terms like nitrocellulose membrane), the false positive rate increased.

## Discussion

Our results were more convincing for protein entity extraction than for GO-term extractions. This, we suggest, is the result of two principal factors. One is the lexical properties of protein names and symbols considered as proper names, which, often display string features which are easier to detect, such as capital letters and special characters; while GO-terms are adverbial nouns, often lacking such characteristics. This could explain the higher entity extraction achieved for proteins. Another reason could be the limited number of synonyms and natural language variants of proteins when compared to GO-terms. In other words, there are fewer alternatives for expressing a protein entity in free text, while GO-terms can be reformulated in a broad variety of ways, often not even in the form of continuous text segments.

Our system is especially useful for the extraction of molecular function annotations for proteins; while in the case of biological process annotations it could still be improved, as the extraction of GO entities from this category is still rather low.

**Figure 1:**
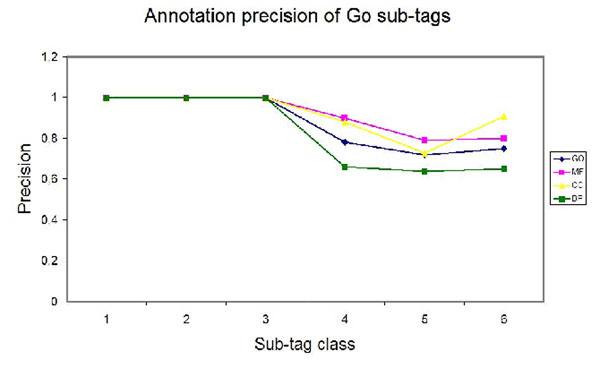
**Annotation precision of GO sub-tags. **The precision of each sub-tag for the GO-term entity is displayed. GO: all GO categories, MF: Molecular Function category, CC: Cellular Component category, BP: Biological Process category. The sub-tag classes correspond to: 1 original GO-term, 2 natural language variants of the GO-term, 3 externally linked terms and synonyms, 4 GO-term forming words, 5 GO-term definition forming words and 6 are GO-term co-occurring words in PubMed sentences after extended stop word filtering.

Aside from increasing system speed and offering alternative sliding window sizes, among the potential refinements are the use of different sub-tag scoring schemes. A statistical analysis of the precision of each sub-tag set revealed significant differences in sub-tag precisions depending on GO-category (see figure 1). Thus a distinct score for each sub-tag based on its precision and depending on the corresponding GO category might be useful. For instance, in the case of sub-tag 4 (word types extracted from GO-term definitions), the precision for the category Biological Process is considerably lower than the other two categories.

As the dataset used to derive the sub-tag scores (GOA dataset, see material and methods section) was very noisy, it was diffcult to perform statistically significant analysis using common NLP methods. Among the sources of noise encountered in this dataset were the different annotation conventions depending on the background knowledge of the annotator. Further, major changes in curation conventions over time might have influenced the annotation extraction criteria used for the manual annotations. The most significant problem was the fact that the actual annotations were performed using full length articles. As we only had access to document abstracts, whilst the passage of text relevant for annotation extraction will often be located in other parts of an article (e.g. tables or in the results section), some of the abstracts might even lack the text segments relevant for annotation. Therefore some of the sub-tag weights used did not correlate with their precision.

**Figure 2:**
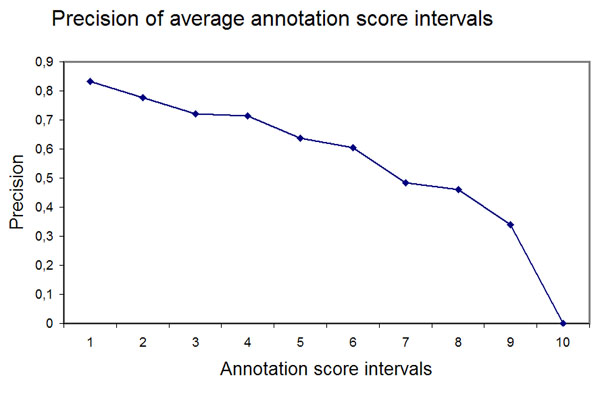
**Precision of average annotation score intervals. **The precision of each annotation score interval is presented. Interval 1: > 90001, 2: 70001–80000, 3: 60001–70000, 4: 50001–60000, 5: 40001–50000, 6: 30001–40000, 7: 20001–30000, 8: 10001–20000, 9: 5001–10000, 10: 0–5000.

**Figure 3:**
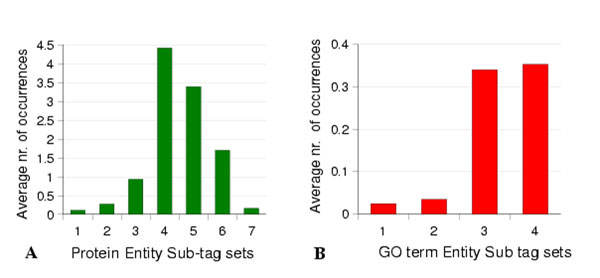
**Average occurrences of members of each sub-tag set within GOA abstract sentences. **A) Protein name sub-tags, 1: original gene name provided by GOA, 2: heuristic typographical variants of the gene name, 3: variants extracted from links to external databases, 4: word types which build up the gene names, 5: word types which build up the external linked gene names, 6 and 7: GOBO sequence ontology and mutation event terms respectively. B) GO-sub-tags, 1: original GO-term, 2: NL-variant of GO-term, 3: word types which build up the GO-term, 4: word types which build up the GO-term definitions. Note that not all the categories are displayed in the bar diagram, co-occurring word types for GO-terms which were extracted from PubMed sentences have an average occurrence in GOA abstract sentences of 11.3337254243.

**Figure 4:**
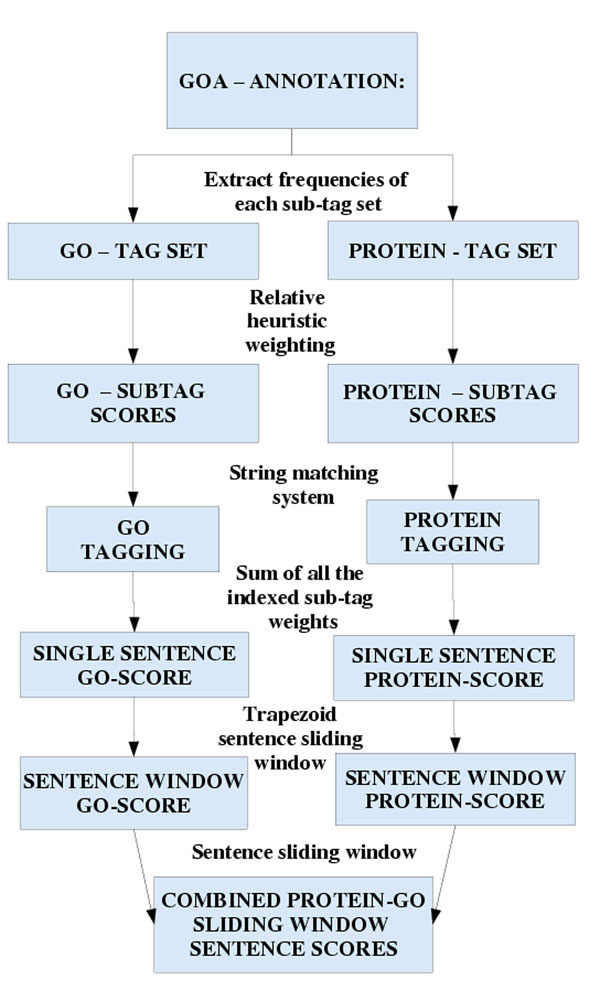
**Scheme illustrating the combination of the distinct entity sentence scores. **A: Trapezoid sentence sliding window generates the average sentence scores for each entity using the sub-tag scores of the matched words (document entity profiles), B: The average sentence scores of each entity are used to generate the combined average annotation score using a second step sliding window (document annotation profile), C: Selection of the highest combined average annotation score, D: Return sentences corresponding to the sentence window with the highest combined average annotation score.

**Figure 5:**
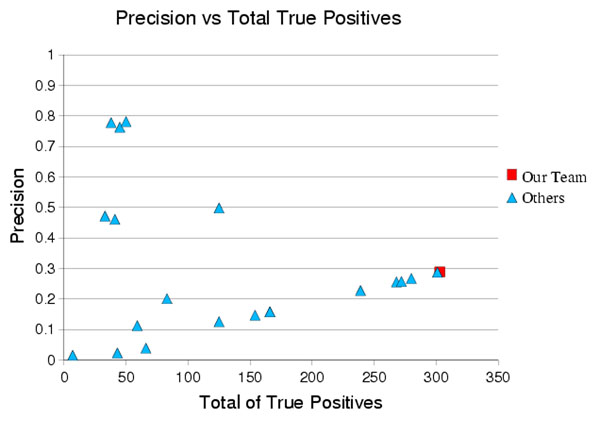
**True positives versus precision. **This figure shows the performance of our system in terms of the total of true positives submitted versus the obtained precision.

The annotation score may serve to determine the confidence intervals of the distinct predictions (see figure 2). Thus depending on the obtained annotation score it is possible to estimate the reliability of the automated annotation extraction. Correct predictions correlate with higher annotation scores while bad predictions tend to score significantly lower.

We believe that including contextual sentence patterns could be useful to improve annotation extraction techniques. Those patterns are based on verbs which occur in sentences describing functional aspects of proteins. Some initial steps have been made in this direction in the form of the automatic extraction of sentence patterns [28], but there was no evaluation using curator based annotations. The sliding sentence window method proposed here was able to score text passages based on contextual information about whether they contain relevant information for a given entity. Combining the window scores allows the merging of individual entity extraction into relation (annotation) extraction. The use of annotation scores provided by the sliding window could provide confidence intervals in terms of the precision of predicted annotations. The optimization of the scoring scheme based on detailed statistical analysis of each sub-tag set might be useful to enhance this system. Also flexible windows size and alternative combinations of the entity scores might improve the performance. We believe that, with such improvements, these preliminary steps could lead to a method useful for real world applications. We are also planning to apply this strategy in the context of gene expression array data.

## Conclusion

Our results demonstrate how contextual information could be exploited to extract protein annotations using full text articles through the use of sentence sliding windows. Our approach was validated at the BioCreative evaluation, which allowed additional performance comparison with alternative techniques. This system performed better for individual entity extraction's (GO-term and protein extraction) when compared with annotation extraction's (Protein-GO-term relation extraction).

This sentence sliding window method is able to score sentence windows of full text articles relative to the given query entities such as proteins and GO terms, as well as annotations based on those entities in cases were a standard training set is missing.

## Authors' contributions

MK and MP conceived the initial idea, implemented it and performed the low level processing steps and statistical evaluations and AV supervised and coordinated the project. MK and AV authored the manuscript.

## Figures and Tables

**Table 1:** Result summary for task 2a. The table shows the percentages of evaluated evidences organized by precision categories for proteins (rows) versus precision categories of GO-terms (columns). The label corresponds to, high: correct prediction, general: not totally wrong prediction but too general to be really useful for protein annotation (for GO-terms) and that the specific protein is not there but a homologue from another organism or a reference to the protein family is contained (for Protein), low: means basically wrong. Total refers to the entity extraction (protein or GO-term) and None are not evaluated cases.

Entity evaluations	GO Low	GO General	GO High	GO None	Total
Protein High	221 (21.05%)	69 (6.57%)	303 (28.85%)	1 (0%)	594 (56.47%)
Protein General	47 (4.48%)	24 (2.28%)	112 (10.67%)	0 (0%)	183 (17.43%)
Protein Low	127 (12.10%)	43 (4.10%)	86 (8.19%)	0 (0%)	256 (24.39%)
Protein None	1 (0.10%)	0 (0%)	0 (0%)	17 (1.61%)	18 (1.71%)
Total	396 (37.73%)	136 (12.95%)	501 (47.71%)	17 (1.61%)	1050 (100%)

**Table 2:** Entity extraction performance of GO-terms. The table shows the evaluated evidences organized by precision categories for the GO-term entity. The label corresponds to, high: correct prediction, general: not totally wrong prediction but too general to be really useful for annotations, low: means basically wrong. MF: Molecular function category, CC: Cellular Component category, BP: Biological Process category.

GO category	Low	General	High	None	Total
MF	103 (30.29%)	40 (11.76%)	178 (52.36%)	19 (5.59%)	340
CC	52 (28.11%)	27 (14.59%)	104 (56.22%)	2 (1.08%)	185
BP	240 (43.72%)	72 (13.11%)	218 (39.71%)	19 (3.46%)	549

**Table 3:** Entity sub-tag scheme. The basic scheme of sub-tag list for the GO entity class and the protein entity class. Each sub tag list containd word types, names or terms with a certain relation to the actual query entity.

GO sub-tag set	Protein sub-tag set
GO-term (original)	Protein name / symbol
NL-GO-term	Variants of Protein name
Externally linked terms	Externally linked names
GO word tokens	Protein name word tokens
GO definition tokens	GOBO mutation term
GO co-occurence tokens	GOBO sequence term
